# Association between oral dysbiosis and Parkinson’s disease: a systematic review

**DOI:** 10.3389/fcimb.2025.1564362

**Published:** 2025-05-13

**Authors:** Laura Murcia-Flores, Ana Sánchez-García, María Pilar Pecci-Lloret, Francisco Javier Rodríguez-Lozano

**Affiliations:** ^1^ Department of Health Sciences, Catholic University San Antonio of Murcia, Murcia, Spain; ^2^ Dermatology, Stomatology, Radiology and Physical Medicine, Hospital Morales Meseguer, Medicine School, University of Murcia, Murcia – Biomedical Research Institute (IMIB), Murcia, Spain

**Keywords:** Parkinson, disease, oral dysbiosis, neurodegenerative, systematic review

## Abstract

The oral cavity serves as the gateway to the human organism, hosting a diverse community of microorganisms that coexist in a state of symbiosis. Disruption of this balance leads to oral dysbiosis, a condition associated with infections and oral pathologies, which may contribute to the etiopathogenesis of systemic disorders such as Parkinson’s disease, a neurodegenerative movement disorder characterized by resting tremor, rigidity, and bradykinesia. While oral dysbiosis is recognized as a risk factor and an aggravating element for Parkinson’s disease, it is not regarded as a direct cause. This systematic review aims to synthesize existing research exploring the potential relationship between oral dysbiosis and the development of Parkinson’s disease. Following a comprehensive analysis, 12 studies were selected, comprising 11 case-control studies and one observational analytical study. These studies investigated the composition of oral microbiota in different sample groups, revealing a higher abundance of pathogenic oral bacteria in individuals diagnosed with Parkinson’s disease. The findings suggest that oral dysbiosis may influence both the onset of Parkinson’s disease and the progression of symptoms such as cognitive decline. These results pave the way for future research, particularly regarding alterations in oral microbiota as potential biomarkers for early diagnosis and disease monitoring.

**Systematic review registration:**

https://www.crd.york.ac.uk/prospero/, identifier CRD42024540056.

## 1 Introduction

The oral cavity, often described as the gateway to the body, hosts a diverse microbiota that plays a crucial role in maintaining overall health (Nicholson and Landry, 2022). This complex environment, comprising habitats like the tongue, teeth, soft and hard palates, and cheeks, provides the ideal conditions—characterized by moisture and warmth—for the coexistence of approximately 500 to 700 different microbial species. Under healthy conditions, this microbiota maintains a symbiotic relationship with the human host, contributing to the defense against pathogenic invasions and supporting both oral and systemic health (Asakawa et al., 2024; Kunath et al., 2024).

In a balanced oral ecosystem, commensal species such as Streptococcus mitis, Gemella, Granulicatella, and Veillonella dominate. Streptococcus mitis, in particular, is prevalent across various oral habitats and plays a significant role in forming biofilms on tooth surfaces through its interaction with salivary proteins like α-amylase (Wade, 2013). This biofilm, when in equilibrium, functions as a protective barrier against external pathogens. However, disturbances in this delicate balance—whether through changes in salivary flow, dietary habits, medication intake, or aging—can lead to dysbiosis. In oral dysbiosis, there is an increase in pathogenic species and a decrease in beneficial species (Song et al., 2022). This shift in the microbial composition often results in the proliferation of pathogenic species such as Porphyromonas gingivalis and Streptococcus mutans, which are associated with oral diseases like periodontitis, caries, and endodontic infections (Asakawa et al., 2024; Chung et al., 2024).

Recent studies have highlighted the potential link between oral dysbiosis and systemic conditions, particularly neurodegenerative diseases such as Parkinson’s disease (PD) (Nicholson and Landry, 2022). This relationship is increasingly evident as research uncovers distinct differences in the oral microbiota of patients with Parkinson’s compared to healthy individuals. The pathogenesis of Parkinson’s, a disorder characterized by motor dysfunction and attributed to the degeneration of dopaminergic neurons in the substantia nigra, may be influenced by factors such as chronic inflammation and microbial infections (Tao et al., 2024). Notably, the presence of pathogens like Porphyromonas gingivalis, known for its role in periodontal disease, has been implicated in promoting systemic inflammation and potentially contributing to neurodegenerative processes (Olsen et al., 2020; Goyal et al., 2023; Li et al., 2024), this is due to its ability to secrete gingipains, which degrade essential neuronal proteins, contributing to the disruption of the blood-brain barrier (Nonaka et al., 2022; Li et al., 2024). Therefore, it is believed that the oral microbiota may influence neuroinflammation (Ranjan et al., 2018), in addition to being closely related to the gut microbiota, which also influences this type of disease (Alam et al., 2024; Costa et al., 2024).

Given the emerging evidence linking oral microbiota and neurodegenerative diseases, this systematic review aims to explore the association between oral dysbiosis and if it contributes to the development and progression of Parkinson’s disease by influencing neuroinflammatory and neurodegenerative pathways. Understanding this connection could pave the way for new preventative and therapeutic strategies, highlighting the importance of oral health in managing systemic conditions. Furthermore, this review seeks to bridge the gap in current research by focusing on the oral microbiome, an area that, despite its significance, has received less attention compared to the gut microbiome in the context of neurodegenerative diseases.

## 2 Methods

This systematic review was conducted in accordance with the PRISMA 2020 guidelines, which stand for “Preferred Reporting Items for Systematic Reviews and Meta-Analyses” (Liberati et al., 2009). Additionally, the review was registered with the PROSPERO database (CRD42024540056) to ensure transparency and avoid duplication of similar reviews (Schiavo, 2019). Additionally, the PCO model (Santos et al., 2007)was used to formulate the following research question:

Is there an association between oral dysbiosis and Parkinson’s disease? (P: Patients/animals with Parkinson’s disease; C: Healthy patients; O: oral pathogens involved in Parkinson’s disease).

The search strategy, study selection, data extraction, and quality assessment (including the risk of bias evaluation) were conducted independently by two investigators (A.S.G. and F.J.R.L.). Any disagreements or uncertainties during the process were resolved through consultation with a third investigator (L.M.F.).

### 2.1 Search strategy

The search terms used to retrieve articles were derived from the MeSH (Medical Subject Heading) thesaurus. The terms related to oral microbiota included: “oral bacterium,” “microorganisms,” “periodontal pathogens,” “oral pathogens,” while those associated with the disease of interest were: “Parkinson disease” and “Parkinson.” Boolean operators (“AND” “OR,” and “NOT”) were employed to link these terms effectively. The search was conducted in February 2024.

### 2.2 Inclusion and exclusion criteria

The inclusion and exclusion criteria are presented in **Table 1** and were established based on the research question and study objectives.

**Table 1:** Inclusion and exclusion criteria.

Inclusion criteria	Exclusion criteria
Articles that examined the potential impact of oral microbiota and its dysbiosis on the development of Parkinson’s disease.	Articles that focused on microorganisms outside the oral cavity, such as gut microbiota (e.g., Helicobacter pylori) or fecal microbiota or articles studies addressing other diseases like Alzheimer’s or multiple sclerosis.
Articles in English or Spanish.	Articles in a language other than English or Spanish.
Observational studies (case-control, cohort, cross-sectional, longitudinal).	Clinical cases, systematic reviews, and meta-analyses.
Studies conducted in humans or animals.	*In vitro* studies.

### 2.3 Study selection

The references retrieved from the search were imported into the citation management software EndNote (Clarivate Analytics, London, United Kingdom) to identify and eliminate any duplicates. An initial screening was conducted by examining the titles, followed by a review of the abstracts according to the predefined inclusion and exclusion criteria. Articles that satisfied these criteria were then subjected to a full-text review to determine their eligibility for inclusion in the qualitative synthesis.

### 2.4 Study data

For the bibliometric analysis, the following information was recorded for each article: author and year of publication, journal, and country of publication. Additionally, a table was created to summarize the following data: author, year of publication, study design, sample size and group, type of oral microbiota, method of microbiota extraction, and the association between Parkinson’s disease and oral dysbiosis, if any.

### 2.5 Quality assessment

The quality of the articles included in this systematic review was evaluated using a modified version of the STROBE (Strengthening the Reporting of Observational Studies in Epidemiology) checklist (von Elm et al., 2008) (**Table 2**). The checklist is primarily applied to cohort studies, case-control studies, and cross-sectional studies, and it assesses various aspects of study quality across 10 key points, such as participants, variables, data sources, and statistical methods. The risk of bias in each study was categorized as low, moderate, or high based on the number of checklist points fulfilled: low risk (8–11 points), moderate risk (5–7 points), and high risk (1–4 points). Each checklist item was marked with a tick if the requirement was met or with a cross if it was not.

**Table 2:** List of criteria used to evaluate the quality of observational studies based on an adapted version of the STROBE guidelines.

Methods		
Configuration	1	Describe the environment, locations, and relevant dates, including the periods of recruitment, exposure, follow-up, and data collection.
Participants	2	Specify the eligibility criteria (inclusion and exclusion), including matched groups or control if applicable.
	3	Describes the disease studied
Variables	4	Presence the oral microbiota.
Data Sources/Measurement	5	Provide a detailed explanation of the evaluation methods (isolation) of the oral microbiota.
Study Size	6	Explain how the study size was determined.
Statistical Methods	7	Describe all statistical methods, including those used to control confounding factors.
	8	Describe any method used to examine subgroups and interactions.
Descriptive Data	9	Provide the characteristics of the study participants (e.g., demographic, clinical, social), and report on exposures and potential confounding factors.
Outcome Data	10	Measures and presents exposure data.

## 3 Results

### 3.1 Study selection and flow diagram

**Figure 1:**
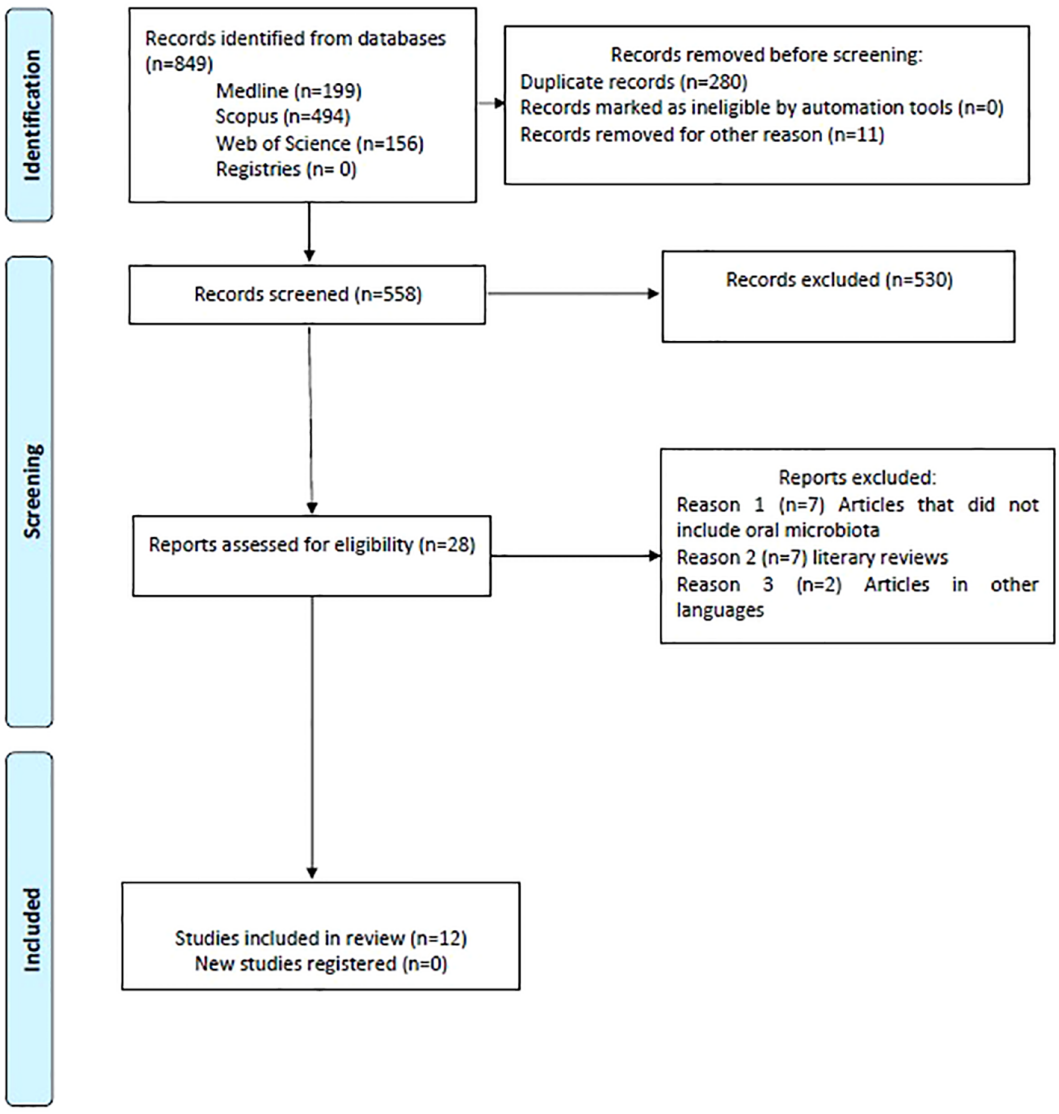
Flow diagram.

Following a comprehensive and meticulous database search, a total of 849 references were identified: 199 from PubMed, 494 from Scopus, and 156 from Web of Science. The results obtained from each database are summarized in **Table 3**. These references were imported into the Endnote, where 280 duplicates, including undetected ones, were removed. The remaining 558 articles underwent screening based on their titles and abstracts, leading to the exclusion of 530 articles. This left 28 articles for full-text review. Upon further examination, 7 articles were excluded for not addressing oral microbiota, 2 were excluded due to being published in Chinese, and 7 were excluded for being systematic reviews. Consequently, 12 articles were selected for inclusion in this review (**Figure 1**).

**Table 3:** Results obtained from each database.

Database	Search Field	Results
PubMed	1# “oral bacterium” OR “microorganisms” OR “periodontal pathogens” OR “oral pathogens”	152,500
	2# “parkinson disease” OR “parkinson”	157,169
	1# AND 2#	199
Scopus	1# “oral bacterium” OR “microorganisms” OR “periodontal pathogens” OR “oral pathogens”	757,461
	2# “parkinson disease” OR “parkinson”	208,052
	1# AND 2#	494
Web of Science	1# “oral bacterium” OR “microorganisms” OR “periodontal pathogens” OR “oral pathogens”	205,243
	2# “parkinson disease” OR “parkinson”	165,779
	1# AND 2#	156

### 3.2 Characteristics of the studies

#### 3.2.1 Bibliometric analysis

**Figure 2:**
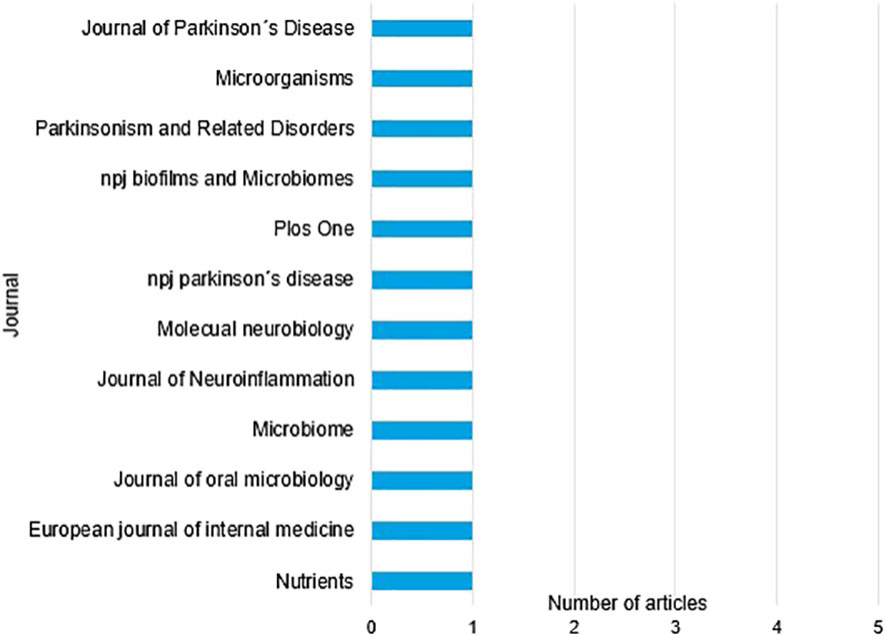
Organization of articles by year of publication.

**Figure 3:**
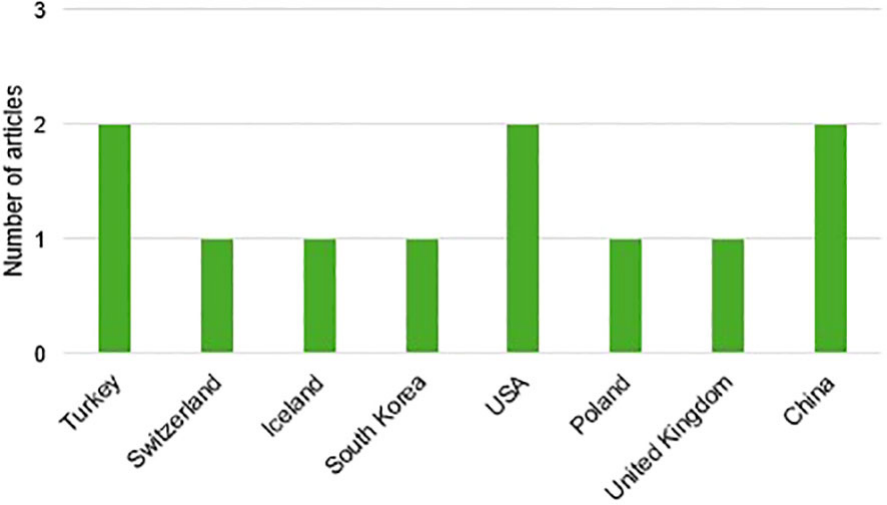
Organization of articles by country of publication.

**Figure 4:**
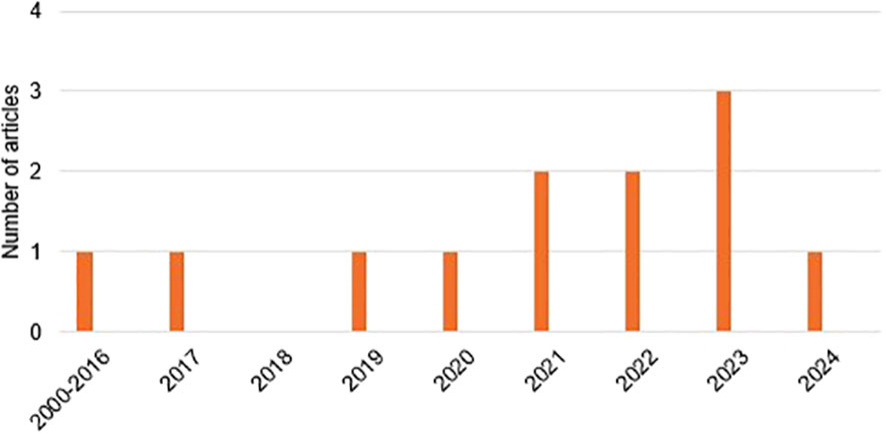
Organization of articles by journal of publication.

The distribution of the selected articles by year of publication is shown in **Figure 2**, where we can observe that the year with the highest prevalence of studies was 2023 with three studies, followed by 2022 and 2021 with two studies each. The distribution by country in **Figure 3**, with Turkey, the USA, and China being the countries with the highest number of articles, with two each. Finally,and by journal in **Figure 4**, with all journals having a single publication.

#### 3.2.2 Study design

All the selected articles are case-control studies, where healthy subjects are compared with those diagnosed with Parkinson’s disease, except for the oldest study conducted by Gosney et al. (Gosney et al., 2003), in which all subjects have Parkinson’s disease. This particular study is an observational analytical study. Additionally, it is important to note that, among the selected studies, all were conducted on human subjects, except for two studies that were performed on mice.

#### 3.2.3 Oral microbiota

As shown in **Table 4**, the genera with the highest abundance among Parkinson’s patients are, firstly, the genus *Streptobacillaceae*, cited in six studies, with specific mention of bacteria such as *S. mutans* and *S. anginosus*, which are significantly relevant to the subject under discussion. Some studies also refer to other types, such as *S. sanguinis* or *S. sobrinus*, although these are of lesser relevance (Zapala et al., 2022). The families *Lactobacillaceae* and *Prevotellaceae* are also frequently cited, appearing in the results of five of the analyzed studies. The latter is noted for an abundance of bacteria such as *P. intermedia*, *P. histicola*, and *P. melaninogenica*. Additionally, three studies highlight the genus *Veillonellaceae (* Pereira et al., 2017; Fleury et al., 2021; Bai et al., 2023). Notably, however, only two studies identified *P. gingivalis* as the primary pathogen (Feng et al., 2020; Li et al., 2024). The remaining genera have been identified as abundant in the oral microbiota of these patients in only one study.

**Table 4:** Results: main characteristics of the studies included.

AUTHOR	TYPE OF STUDY	GROUP SAMPLE	ORAL MICROBIOTE	OBTAINING MICROBIOTA METHOD	ASSOCIATION
Dragos Mihaila et al. (2019)	Case and controls	N=84 (N1 = 48 Parkinson N2 = 36 control)	*Lactobacillus*, *Bifidobacterium*, *Candida, Saccharomyces* *cerevisiae*	Saliva samples by expectoration in a vial expectoration in an OraGene RNA OraGene RNA collection vial.	Yes
Xue Bing Bai et al. (2023)	Animals study	N = male mice N1= control N2= periodontitis N3= Parkinson (3.6 Tetrahidropiridine) N4: Parkinson and periodontitis	*Veillonella parvula*, *S.mutans*	Subgingival plaque samples from patients with periodontitis applied to the gingival tissue of mice.	Yes
Pedro A.B. Pereira et al. (2017)	Case and controls	N=152 (n1 = 72 Parkinson) (N2 = 76 control)	*Streptococcus*, *Haemophilus, Neisseria*, *Veillonella and Prevotella*	Brushing of buccal mucosa and sublingual mucosa with cotton swabs.	Yes
Natalia Rozas et al. (2021)	Case and controls	N=60 (N1 = 30 Parkinson) (N2 = 30 control)	*Lactobacillus, T.forsythia and* *P. intermedia*	Catch-All sample collection swabs Catch-All swabs, in hard and and soft tissue.	Yes
Vanessa Fleury et al. (2021)	Case and controls	N=40 (N1 = 20 Parkinson) (N2 = 20 control)	*S. mutans* *Kingella oralis*, *Actinomyces*, *Veillonella*	Saliva: Sterile plastic tube Subgingival plaque: Sterile paper sterile paper tip in the deepest pocket. deepest pocket.	Yes
Sangyang Jo et al. (2022)	Case and controls	N=143 (N1 = 74 Parkinson) (N2 = 69 control)	*Lactobacillus*	Saliva samples for 16S rRNA gene analysis	Yes
Barabara Zapala et al. (2022)	Case and controls	N=167 (N1 = 59 Parkinson) (N2 = 108 control)	*Prevotella, Streptococcus*, *and lactobacillus*	Tongue, hard palate and mucosa samples with swabs	Yes
Muzaffer Arikan et al. (2023)	Case and controls	N=115 (N1 = 45 Parkinson mild cognitive impairment) (N2 = 43 Parkinson’s with dementia) (N3 = 27 control)	*S. mutans, Prevotella*, *Veillonella, Campylobacter*, *Neisseria*	Saliva samples used for DNA and protein extraction protein extraction.	Yes
Margot Gosney et al. (2003)	Observational study	N=50 (Parkinson)	*Escherichia coli*, *Klebsiella* spp., *Kluyvera* spp. *Serratia* *spp.Proteus* spp., *Enterobacter* spp.	Dry sample from the oral cavity cavity. It is obtained by moving the swab around the area of tonsils and the soft palate palate	Yes
Ekin Yay et al. (2023)	Case and controls	N=60 (N1 = 20 Parkinson and periodontitis) (N2 = 20 Periodontitis) (N3 = 20 control)	*Treponema socranskii*, *Peptostreptococcaceae*, *Parvimona micra*, *Lachnoanaerobaculum* *saburreum*, *Prevotella melaninogenica*, *Streptococcus anginosus*,	Subgingival plaque samples from uniradicular teeth.	Yes
Dongcheng Li et al. (2024)	Case and controls	N=50 (N1 = 20 EP mild cognitive impairment) (N2 = 15 Parkinson normal cognitive) (N3 = 15 control)	*P. gingivalis*	Collection of gingival crevicular gingival crevicular fluid.	Yes
Yu Kun Feng et al. (2020)	Animal study	N=40 mice	*P. gingivalis*	Administration of live P. gingivalis live by oral administration to animals three times a week for week for 1 month.	Yes

### 3.3 Quality analysis

The analysis shows a moderate to low risk of bias, with 8 articles rated as low risk and 4 as moderate risk. Notably, no studies were found to have a high risk of bias (**Table 5**). Key findings include that all articles fulfilled item 5 (data sources and measurements), item 1 (study design), and item 3 (participant description). Additionally, all studies provided exposure data (item 10) and clearly defined diagnostic criteria (item 4). However, only one article, by Zapała et al (Zapala et al., 2022), met item 6 (sample size calculation), and just 5 articles detailed the statistical methods, including subgroups and interactions. One article, by Gosney et al (Gosney et al., 2003), had the highest risk of bias, scoring 5 points, due to the absence of inclusion/exclusion criteria, sample size details, and statistical analysis with subgroups, along with a lack of participant characteristic descriptions.

**Table 5:** Results of the quality assessment conducted with an adapted version of the STROBE guidelines.

	Mihaila Gosney et al. (2003)	Bai et al. (2023)	Pereira et al. (2017)	Rozas et al. (2021)	Fleury et al. (2021)	Jo et al. (2022)	Zapala et al. (2022)	Arikan et al. (2023)	Gosney et al. (2003)	Yay et al. (2023)	Li et al. (2024)	Feng et al. (2020)
**1**	✓	✓	✓	✓	✓	✓	×	✓	✓	✓	✓	✓
**2**	✓	×	✓	✓	✓	✓	✓	✓	×	✓	✓	×
**3**	✓	✓	✓	✓	✓	✓	✓	✓	✓	✓	✓	✓
**4**	✓	✓	✓	✓	✓	✓	✓	✓	✓	✓	✓	✓
**5**	✓	✓	✓	✓	✓	✓	✓	✓	×	✓	✓	✓
**6**	×	×	×	×	×	×	✓	×	✓	×	×	×
**7**	×	✓	✓	✓	✓	✓	×	✓	×	✓	✓	✓
**8**	×	×	×	✓	✓	×	×	✓	×	✓	×	✓
**9**	✓	×	✓	✓	✓	✓	✓	✓	×	✓	✓	×
**10**	✓	✓	✓	✓	✓	✓	✓	✓	✓	✓	✓	✓
**Total Score**	7	6	8	9	9	8	8	9	5	9	8	10
**Risk of bias**	Moderate	Moderate	Low	Low	Low	Low	Low	Low	Moderate	Low	Low	Low

## 4 Discussion

As the global population continues to age, the prevalence of Parkinson’s disease (PD) is rising, particularly among the elderly, with cognitive impairment recognized as a critical non-motor symptom (Mosaddad et al., 2023). PD is the second most prevalent neurological disorder, affecting approximately 1% of individuals over 60 and between 4% and 5% of those over 80. The incidence is 1.5 times higher in males than females. Despite genetic variations being implicated in less than 10% of Parkinson’s cases, environmental risk factors have emerged as an area of significant research interest (Zapala et al., 2022). The complex relationships between aging, genetic predispositions, and environmental factors are thought to underlie the still-unclear etiology of PD. Emerging evidence suggests that certain pathogenic bacteria within the oral microbiome may contribute to cognitive decline and the progression of Parkinson’s disease (Rozas et al., 2021; Tao et al., 2024).

Studies have demonstrated that the oral microbiota plays a crucial role in both local and systemic health, interacting with a variety of oral and systemic diseases. In older adults, physiological changes can disrupt the balance between pathogenic and commensal bacteria, potentially increasing pathogenic gene expression and disease susceptibility (Sampaio-Maia et al., 2016; Kozak and Pawlik, 2023). The common occurrence of tooth loss in this age group further disrupts the oral microbial environment by removing habitats essential for certain bacteria, such as subgingival sites and tooth surfaces. Additionally, dentures alters the oral microbiome by impacting bacterial quantity and diversity. The frequent occurrence of oral diseases in older adults suggests that these age-associated changes in the oral microbiome may play a role in the development of such conditions. This hypothesis is supported by research linking age-related microbial shifts (Preza et al., 2009; Liu et al., 2020) to the onset of systemic diseases prevalent among the aging population (Soliman et al., 2022).

The relationship between the oral cavity and the brain has garnered increasing attention, particularly regarding the oral-gut-brain axis and its role in neurological diseases (Laugisch et al., 2024). Previous research has demonstrated that gut microbiota can communicate with the brain via the gut-brain axis, influencing neurodevelopmental processes. The gut and oral microbiota are susceptible to various host-related factors, which can, in turn, make the brain more vulnerable to dysfunction (Ma et al., 2024). While research specifically addressing oral-brain communication is still limited, emerging hypotheses suggest that the oral microbiota-brain axis could impact neural activity, similar to the way aroma release and perception are influenced (Chen et al., 2023).

Unlike gut microbiota, the oral microbiota is distinct and can enter the gastrointestinal tract through saliva, thereby altering the intestinal microbial community (Qian et al., 2023). This translocation can trigger inflammation-related changes and modulate the immune system (Socala et al., 2021). The movement of oral bacteria to the gut is a characteristic feature of certain diseases, and the transmission of oral microbes via fecal matter can significantly shape the gastrointestinal microbiome. Moreover, studies suggest that the oral cavity may act as a reservoir for intestinal pathogens, which have the potential to activate the intestinal immune system and contribute to chronic inflammation (Goyal et al., 2023).

Numerous hypotheses have been proposed regarding the bacterial family Lactobacillaceae in relation to Parkinson’s disease. Jo et al. (2022) suggest that *L. reuteri*, present in the mouth and gut, may increase the release of α-synuclein in the enteric nervous system, potentially leading to gut colonization by pathogens. Similarly, Mihaila et al. (2019) found that this pathogen is significantly elevated in individuals with Parkinson’s disease and is positively correlated with slowed movement. On the other hand, Zapala et al. (2022) highlighted that, although the genus *Prevotellaceae* is a common resident in the oral cavity, it can occasionally become pathogenic, causing intestinal inflammation and contributing to systemic inflammation, which is implicated in various diseases, including Parkinson’s. They identified *P. histicola, P. melaninogenica*, and *P. gingivalis* as the most abundant bacteria in these patients. However, these findings differ from other studies, such as Pereira et al (Pereira et al., 2017), who reported a greater diversity of bacteria including *Streptococcus, Neisseria, Haemophilus*, and *Veillonella*, and Rozas et al (Rozas et al., 2021), who identified *Lactobacillus, T. forsythia*, and *P. intermedia* as more prevalent. Despite differences, these studies concur that members of the *Prevotellaceae* family increased in the oral cavity of Parkinson’s patients, in contrast to a decrease in this genus in the gut microbiota, which appears to be possibly linked to the oral hygiene of this type of patients (Pereira et al., 2017). This pattern suggests that opportunistic pathogens within this species may be present due to hygiene deficiencies associated with motor and non-motor symptoms (Pereira et al., 2017). Nonetheless, the results regarding the abundance of this genus do not yet provide precise conclusions, and further studies are necessary to confirm the hypothesis that these bacteria could serve as biomarkers, we would need better-designed, standardized studies with proper statistical analysis (Zapala et al., 2022).

Several studies have explored the association between periodontal disease and Parkinson’s disease, focusing on the role of systemic inflammation and the resulting neuroinflammation caused by periodontitis in the development of Parkinson’s. This process is thought to involve changes in the permeability of the blood-brain barrier, microglial activation, and the entry of pathogens into the brain. Li et al. (2024) propose that the pathogenesis of cognitive impairment in neurodegenerative diseases is not entirely clear, but note that patients with such impairments often exhibit oral dysbiosis. They identify *P. gingivalis* as a potential key factor in cognitive decline, particularly due to its lysine-gingipain enzyme. Various studies suggest two possible mechanisms by which *P. gingivalis* could contribute to cognitive deterioration: first, by entering the bloodstream from periodontal pockets, triggering systemic inflammation and β-amyloid production, which disrupts the blood-brain barrier, allowing lipopolysaccharides and gingipain to penetrate the brain and cause neuroinflammation; second, by entering the gut, inducing dysbiosis, inflammation, and abnormal accumulation of α-synuclein, which then spreads to the brain via the vagus nerve (Feng et al., 2020; Laugisch et al., 2024; Li et al., 2024).

Alterations in the oral microbiome have been extensively studied in Alzheimer’s disease, another neurodegenerative disorder, where oral dysbiosis—primarily due to periodontal disease—has been shown to significantly contribute to the formation of β-amyloid deposits in the brain, a major etiopathogenic hallmark of the disease (Sureda et al., 2020; Wan and Fan, 2023). Lipopolysaccharides and other byproducts from periodontitis elevate cerebral pro-inflammatory mediators (IL-1β, TNF-α, IL-6), which promote amyloid accumulation, leading to neurodegeneration and Alzheimer’s disease (Sureda et al., 2020). Similarly, oral dysbiosis is implicated in other systemic diseases, including cancer (Tuominen and Rautava, 2021), cardiovascular pathologies (Tonelli et al., 2023), aspiration pneumonia (Pathak et al., 2021), and diabetes (Xiao et al., 2017). In cancer, bacteria such as *P. gingivalis* and *F. nucleatum* exhibit carcinogenic properties by stimulating cell proliferation, facilitating cellular invasion, and inciting chronic inflammation (Tuominen and Rautava, 2021). In cardiovascular disease, oral dysbiosis influences disease progression through mechanisms such as biofilm formation, platelet aggregation, endothelial dysfunction, and systemic inflammation, with bacteria like *A. actinomycetemcomitans*, *P. gingivalis*, *S. mutans*, and *T. denticola* detected in aortic aneurysms and heart valves, and the first two also present in atherosclerotic lesions (Tonelli et al., 2023). Although diabetes mellitus may alter the composition and diversity of the oral microbiota, its precise impact remains controversial (Xiao et al., 2017).

The primary and significant limitation of this systematic review is the limited number of articles published on the impact of oral microbiota on Parkinson’s disease. Most of the available research focuses on gut microbiota or Alzheimer’s disease, which has been more extensively studied. Additionally, many studies were duplicated due to the search being conducted across multiple databases. Articles that were not in Spanish or English were excluded, as well as those that were systematic or narrative reviews. Another limitation found is the significant heterogeneity in the methodology of the selected studies, as well as in sample size and inclusion criteria, making direct comparison of results more complex. Additionally, the case-control selection allows for an association to be established but not a true causal relationship. Therefore, longitudinal studies are needed to evaluate changes in the composition of the oral microbiota over time and their impact on disease progression.

According to the results of this review, oral dysbiosis appears to play a significant role in the etiopathogenesis of Parkinson’s disease, as well as in the symptom progression, due to the abundance of certain oral bacteria that can negatively impact both the oral and systemic microbiome. This highlights the need for continued research into the oral microbiota as a potential non-invasive biomarker, which could help predict Parkinson’s risk and cognitive decline progression.

## 5 Conclusion

Given the results obtained, which highlight the relationship between the oral microbiota and Parkinson’s disease, it becomes evident how undervalued oral health is in the context of neurodegenerative diseases. Future longitudinal studies are needed to analyze changes in the oral microbiota during the early stages of the disease and to monitor their progression over time. Dental treatment aimed at preventing alterations in the microbiota could serve as a complementary strategy for patients at risk of developing neurodegenerative disorders.

## Data Availability

The original contributions presented in the study are included in the article/supplementary material. Further inquiries can be directed to corresponding author.
